# A Novel Algorithm Combining Finite State Method and Genetic Algorithm for Solving Crude Oil Scheduling Problem

**DOI:** 10.1155/2014/748141

**Published:** 2014-02-18

**Authors:** Qian-Qian Duan, Gen-Ke Yang, Chang-Chun Pan

**Affiliations:** Department of Automation and Key Laboratory of System Control and Information Processing, Shanghai Jiao Tong University, Ministry of Education of China, Shanghai 200240, China

## Abstract

A hybrid optimization algorithm combining finite state method (FSM) and genetic algorithm (GA) is proposed to solve the crude oil scheduling problem. The FSM and GA are combined to take the advantage of each method and compensate deficiencies of individual methods. In the proposed algorithm, the finite state method makes up for the weakness of GA which is poor at local searching ability. The heuristic returned by the FSM can guide the GA algorithm towards good solutions. The idea behind this is that we can generate promising substructure or partial solution by using FSM. Furthermore, the FSM can guarantee that the entire solution space is uniformly covered. Therefore, the combination of the two algorithms has better global performance than the existing GA or FSM which is operated individually. Finally, a real-life crude oil scheduling problem from the literature is used for conducting simulation. The experimental results validate that the proposed method outperforms the state-of-art GA method.

## 1. Introduction

In recent years refineries have to explore all potential cost-saving strategies due to intense competition arising from fluctuating product demands and ever-changing crude prices. Scheduling of crude oil operations is a critical task in the overall refinery operations [1–3]. Basically, the optimization of crude oil scheduling operations consists of three parts [4]. The first part involves the crude oil unloading, mixing, transferring, and multilevel crude oil inventory control process. The second part deals with fractionation, reaction scheduling, and a variety of intermediate product tanks control. The third part involves the finished product blending and distributing process. In this paper, we focus on the first part, as it is a critical component for refinery scheduling operations. Scheduling of crude oil problem is often formulated as mixed integer nonlinear programming (MINLP) models [2, 5, 6]. The solution approaches for solving MINLP can be roughly divided into two categories [7]: deterministic approaches and stochastic approaches. Some deterministic methods have been available for many years [8]. These methods require the prior step of identification and elimination of nonconvexity and decompose the MINLP models into relevant nonlinear programming (NLP) and mixed integer linear programming (MILP) and then these subproblems have to be iteratively solved. The most common algorithms are branch and bound [9], outer-approximation [10], generalized benders decomposition [11], and so forth. Also, some commercial MINLP solvers have been developed for solving the problem at hand optimally [12]. However, the commercial solver can only handle MINLPs with special properties. The other stream of global optimization is the stochastic algorithms, for example, simulated annealing (SA), GA, and their variants [7]. GA proposed by Holland [13], because of their simple concept, easy scheme, and the global search capability independent of gradient information, have been developed rapidly. Much other attention is given to the development of GA for MINLP. For instance, Yokota et al. developed a penalty function that is suitable for solving MINLP problems [14]. Costa and Oliveira also implemented another type of penalty function to solve various MINLP problems, including industrial-scale problems [15]. They also noted that the evolutionary approach is efficient, in terms of the number of function evaluations, and is very suitable to handle the difficulties of the nonconvexity. Going one step further, some mixed coding methods were proposed, which include mixed-coding genetic algorithm [15] and information-guided genetic algorithm (IGA). Ponce-Ortega et al. [16] proposed a two-level approach based on GA to optimize the heat exchanger networks (HENs). The outer level is used to perform the structural optimization, for which a binary GA is used. Björk and Nordman [17] showed that the GA is very suitable to solve a large-scale heat exchanger network.

Obviously, the two different approaches previously discussed have their own advantages and disadvantages. On the one hand, a deterministic approach usually involves considerable algebra and undeviating analysis to the problem itself, whereas the evolutionary approach does not have this property. On the other hand, some deterministic approaches, such as mathematical programming, usually cannot provide practical solutions in reasonable time, whereas the evolutionary approach can generate satisfying solutions. In this work, a novel genetic algorithm which combined the finite state method and GA is proposed to solve crude oil scheduling problem. A MINLP model is formulated based on the single-operation sequencing (SOS) time representation. A deterministic finite automation (DFA) model which captures valid possible schedule sequences is constructed based on the sequencing rules. The initialization and mutation operation of GA is based on the model which builds legal schedules complying with sequencing rules and operation condition. Thus, the search space of the algorithm is substantially reduced as only legal sequence is explored. The rest of the paper is organized as follows: the MINLP model is specified in Section 2.  Section 3 reviews the background of finite state theory. In Section 4, a novel genetic algorithm which combined the finite state method and GA is proposed to solve the MINLP model. A test problem is studied to verify our approach in Section 5. In the last section, conclusive remarks are given.

## 2. Mathematic Model

In this section, the MINLP model of refinery crude oil scheduling problem is described [18]. This problem has been widely studied from the optimization viewpoint since the work of Lee et al. [19]. It consists of crude oil unloading from marine vessels to storage tanks, transfer and blending between tanks, and distillation of crude mixtures. The goal is to maximize profit and meet distillation demands for each type of crude blend (e.g., low sulfur or high sulfur blends), while satisfying unloading and transfer logistics constraints, inventory capacity limitations, and property specifications for each blend. The logistics constraints involve nonoverlapping constraints between crude oil transfer operations.

### 2.1. Sets

The following sets will be used in the model.

-   
*T* = {1,…, *n*} is the set of priority-slots;
-   
*W* is the set of all operations: *W*≜*W*_*U*_ ∪ *W*_*T*_ ∪ *W*_*D*_;
-   
*W*_*U*_ ⊂ *W* is the set of unloading operations;
-   
*W*_*T*_ ⊂ *W* is the set of tank-to-tank transfer operations;
-   
*W*_*D*_ ⊂ *W* is the set of distillation operations;
-   
*R* is the set of all operations: *R* = *R*_*V*_ ∪ *R*_*S*_ ∪ *R*_*C*_ ∪ *R*_*D*_;
-   
*R*_*V*_ ⊂ *R* is the set of vessels;
-   
*R*_*S*_ ⊂ *R* is the set of storage tanks;
-   
*R*_*C*_ ⊂ *R* is the set of charging tanks;
-   
*R*_*D*_ ⊂ *R* is the set of distillation units;
-   
*I*_*r*_ ⊂ *W* is the set of inlet transfer operations on resource *r*;
-   
*O*_*r*_ ⊂ *W* is the set of outlet transfer operations on resource *r*;
-   
*C* is the set of products (i.e., crudes);
-   
*K* is the set of product properties (e.g., crude sulfur concentration).

### 2.2. Parameters

Parameters used in the paper are defined below:

- *H* is the scheduling horizon;
- $\left[\underline{V_{v}^{t}},\bar{V_{v}^{t}}\right]$ are bounds on the total volume transferred during transfer operation *V*; in all instances, $\underline{V_{v}^{t}}=0$ for all operations except unloading for which $\underline{V_{v}^{t}}=\bar{V_{v}^{t}}$ is the volume of crude in the marine vessel;
- $\left[\underline{N_{D}},\bar{N_{D}}\right]$ are the bounds on the number of distillations;
- $\left[\underline{{FR}_{v}},\bar{{FR}_{v}}\right]$ are flow rate limitations for transfer operation *v*;
- *S*_*r*_ is the arrival time of vessel *r*;
- $\left[\underline{x_{vk}},\bar{x_{vk}}\right]$ are the limits of property *k* of the blended products transferred during operation *v*;
- *x*_*ck*_ is the value of the property *k* of crude *c*;
- $\left[\underline{L_{r}^{t}},\bar{L_{r}^{t}}\right]$ are the capacity limits of tank *r*;
- $\left[\underline{D_{r}},\bar{D_{r}}\right]$ are the bounds of the demand on products to be transferred out of the charging tank *r* during the scheduling horizon;
- *G*_*c*_ is the gross margin of crude *c*.

### 2.3. Variables

#### 2.3.1. Assignment Variables

(1) $$\begin{array}{c} Z_{iv}\in \left\{0,1\right\},\;i\in T,\;\;v\in W. \end{array}$$

*Z*_*iv*_ = 1 if operation *v* is assigned to priority-slot *i*;  *Z*_*iv*_ = 0 otherwise.

#### 2.3.2. Time Variables

(2) $$\begin{array}{c} S_{iv}\geq 0,\;D_{iv}\geq 0,\;i\in T,\;\;v\in W. \end{array}$$

*S*_*iv*_ is the start time of operation *v* if it is assigned to priority slot *i*; *S*_*iv*_ = 0 otherwise.

*D*_*iv*_ is the duration of operation *v* if it is assigned to priority slot *i*; *D*_*iv*_ = 0 otherwise.

#### 2.3.3. Operation Variables

(3) $$\begin{array}{c} V_{iv}^{t}\geq 0,\;V_{ivc}\geq 0,\;i\in T,\;\;v\in W,\;\;c\in C. \end{array}$$

*V*_*iv*_^*t*^ is the total volume of crude transferred during operation *v* if it is assigned to priority slot *i*; *V*_*iv*_^*t*^ = 0 otherwise.

*V*_*ivc*_ is the volume of crude *c* transferred during operation *v* if it is assigned to priority slot *i*; *V*_*ivc*_ = 0 otherwise.

#### 2.3.4. Resource Variables

(4) $$\begin{array}{c} L_{ir}^{t},L_{irc},\;i\in T,\;\;r\in R,\;\;c\in C. \end{array}$$

*L*_*ir*_^*t*^ is the total accumulated level of crude in tank *r* ∈ *R*_*S*_ ∪ *R*_*C*_ before the operation was assigned to priority-slot *i*.

*L*_*irc*_ is the accumulated level of crude *c* in tank *r* ∈ *R*_*S*_ ∪ *R*_*C*_ before the operation was assigned to priority-slot *i*.

### 2.4. Objective Function

The objective is to maximize the gross margins of the distilled crude blends. Let *G*_*c*_ be the individual gross margin of crude *c*,

(5) $$\begin{array}{c} \max\sum_{i\in T\;}\sum_{r\in R_{D}\;}\sum_{v\in I_{r}\;}\sum_{c\in C}G_{c}\cdot V_{ivc}. \end{array}$$

### 2.5. General Constraints

It should be noted that the crude composition of blends in tanks is tracked instead of their properties. The distillation specifications are later enforced by calculating a posteriori the properties of the blend in terms of its composition. For instance, in the problem, a blend composed of 50% of crude A and 50% of crude B has a sulfur concentration of 0.035 which does not meet the specification for crude mix X nor for crude mix Y.

#### 2.5.1. Assignment Constraints

In the SOS model, exactly one operation has to be assigned to each priority slot,

(6) $$\begin{array}{c} \sum_{v\in W}Z_{iv}=1,\;i\in T. \end{array}$$

#### 2.5.2. Variable Constraints

Variable constraints are given by their definitions. Start time, duration, and global volume variables are defined with big-*M* constraints,

(7) $$\begin{array}{c} S_{iv}+D_{iv}\leq H\cdot Z_{iv},\;i\in T,\;\;v\in W,\\ V_{iv}^{t}\leq \overset{-}{V_{v}^{t}}\cdot Z_{iv},\;i\in T,\;\;v\in W,\\ V_{iv}^{t}\geq \underline{V_{v}^{t}}\cdot Z_{iv},\;i\in T,\;\;v\in W. \end{array}$$

Crude volume variables are positive variables whose sum equals the corresponding total volume variable,

(8) $$\begin{array}{c} \sum_{c\in C}V_{ivc}=V_{iv}^{t}. \end{array}$$

Total and crude level variables are defined by adding to the initial level in the tank all inlet and outlet transfer volumes of operations of higher priority than the considered priority slot,

(9) Lirt=L0rt+∑j∈T,j<i ∑v∈IrVivt−∑j∈T,j<i ∑v∈OrVivt, i∈T,  r∈R,

(10) Lirc=Lorc+∑j∈T,j<i ∑v∈IrVivc−∑j∈T,j<i ∑v∈OrVivc,i∈T,  r∈R,  c∈C.

#### 2.5.3. Sequencing Constraints

Sequencing constraints restrict the set of possible sequences of operations. Cardinality and unloading sequence constraints are specific cases of sequencing constraints. More complex sequencing constraints will also be discussed later.

#### 2.5.4. Cardinality Constraint

Each crude oil marine vessel has to unload its content exactly once. ∑_*i*∈*T*_∑_*v*∈*O*_*r*__ *Z*_*iv*_ = 1, *r* ∈ *R*_*V*_. The total number of distillation operations is bounded by $\underline{N_{D}}$  and $\bar{N_{D}}$ in order to reduce the cost of CDU switches,

(11) $$\begin{array}{c} \underline{N_{D}}\leq \sum_{i\in T\;}\sum_{v\in W_{D}}Z_{iv}\leq \bar{N_{D}}. \end{array}$$

#### 2.5.5. Unloading Sequence Constraint

Marine vessels have to unload in order of arrival to the refinery. Considering two vessels *r*_1_, *r*_2_ ∈ *R*_*V*_,*r*_1_ < *r*_2_ signifies that *r*_1_ unloads before *r*_2_,

(12) $$\begin{array}{c} \sum_{j\in T,j<i\;}\sum_{v\in O_{r_{2}}}Z_{jv}+\sum_{j\in T,j\geq i\;}\sum_{v\in O_{r_{1}}}Z_{jv}\leq 1. \end{array}$$

#### 2.5.6. Scheduling Constraints

Scheduling constraints restrict the values taken by time variables according to logistics rules.

#### 2.5.7. Nonoverlapping Constraint

A nonoverlapping constraint between two sets of operations *W*_1_ ⊂ *W* and *W*_2_ ⊂ *W* states that any pair of operations (*v*_1_, *v*_2_) ⊂ *W*_1_ × *W*_2_ must not be executed simultaneously.

Unloading operations must not overlap,

(13) ∑v∈WU(Siv+Div)≤∑v∈WUSjv+H·(1−∑v∈WUZjv),i,j∈T,  i<j.

Inlet and outlet transfer operations on a tank must not overlap,

(14) ∑v∈Ir(Siv+Div)≤∑v∈OrSjv+H·(1−∑v∈OrZjv),i,j∈T,  i<j,  r∈RS∪RC,∑v∈Or(Siv+Div)≤∑v∈IrSjv+H·(1−∑v∈IrZjv),i,j∈T,  i<j,  r∈RS∪RC.

Although we do not consider crude settling in storage tanks after vessel unloading, it could be included in the model with a modified version of constraint (14) taking into account transition times. We define TR_*V*_  as the transition time after unloading operation *v* ∈ *W*_*U*_ and TR as the maximum transition time, TR = max⁡_*v*∈*W*_*U*__TR_*V*_

(15) ∑v∈Ir(Siv+Div+TRv·Ziv) ≤∑v∈OrSjv+(H+TR)·(1−∑v∈OrZjv).

Constraint (15) is valid in the four possible cases:

(16) (∃v1∈Ir,Ziv1=1) ∧(∃v2∈Or,Zjv2=1)⟹Siv+Div1+TRv1≤Sjv2,(∃v1∈Ir,Ziv1=1) ∧(⋁v2∈Or,Zjv2=1)⟹Siv+Div1≤H+TR−TRv1,(⋁v1∈Ir,Ziv1=0) ∧(∃v2∈Or,Zjv2=1)⟹0≤Sjv2,(⋁v1∈Ir,Ziv1=0) ∧(⋁v2∈Or,Zjv2=0)⟹0≤H+TR.

A tank may charge only one CDU at a time,

(17) ∑v∈Or(Siv+Div)≤∑v∈OrSjv+H·(1−∑v∈OrZjv),i,j∈T,  i<j,  r∈RC.

A CDU may be charged by only one tank at a time,

(18) ∑v∈Ir(Siv+Div)≤∑v∈IrSjv+H·(1−∑v∈IrZjv),i,j∈T,  i<j,  r∈RD.

To avoid schedules in which a transfer is being performed twice at a time, thus possibly violating the flow rate limitations, constraint (19) is included in the model,

(19) $$\begin{array}{c} S_{iv}+D_{iv}\leq S_{jv}+H\cdot \left(1-Z_{jv}\right),\;i,j\in T,\;\;i<j,\;\;v\in W. \end{array}$$

#### 2.5.8. Continuous Distillation Constraint

It is required that CDUs operate without interruption. As CDUs perform only one operation at a time, the continuous operation constraint is defined by equating the sum of the duration of distillations to the time horizon,

(20) $$\begin{array}{c} \sum_{i\in T\;}\sum_{v\in I_{r}}D_{iv}=H,\;r\in R_{D}. \end{array}$$

#### 2.5.9. Resource Availability Constraint

Unloading of crude oil vessels may start only after arrival to the refinery. Let *S*_*r*_ be the arrival time of vessel *r*,

(21) $$\begin{array}{c} S_{iv}\geq S_{r}\cdot Z_{iv},\;i\in T,\;\;r\in R_{v},\;\;v\in O_{r}. \end{array}$$

#### 2.5.10. Operation Constraints

Operation constraints restrict the values taken by operation and time variables according to operational rules.

#### 2.5.11. Flow Rate Constraint

The flow rate of transfer operation *v* is bounded by $\underline{{\text{FR}}_{v}}$  and $\overset{-}{{\text{FR}}_{v}}$

(22) $$\begin{array}{c} \underline{{\text{FR}}_{v}}\cdot D_{iv}\leq V_{iv}^{t}\leq \overset{-}{{\text{FR}}_{v}}\cdot D_{iv},\;i\in T,\;\;v\in W. \end{array}$$

#### 2.5.12. Property Constraint

The property *k* of the blended products transferred during operation *v* is bounded by $\underline{x_{vk}}$  and $\overset{-}{x_{vk}}$. The property *k* of the blend is calculated from the property *x*_*ck*_ of crude *c* assuming that the mixing rule is linear,

(23) $$\begin{array}{c} \underline{x_{vk}}\cdot V_{iv}^{t}\leq \sum_{c\in C}x_{ck}V_{ivc}\leq \overset{-}{x_{vk}}\cdot V_{iv}^{t},\;i\in T,\;\;v\in W,\;\;k\in K. \end{array}$$

#### 2.5.13. Composition Constraint

It has been shown that processes including both mixing and splitting of streams cannot be expressed as a linear model. Mixing occurs when two streams are used to fill a tank and is expressed linearly in constraint (10). Splitting occurs when partially discharging a tank, resulting in two parts: the remaining content of the tank and the transferred products. This constraint is nonlinear. The composition of the products transferred during a transfer operation must be identical to the composition of the origin tank,

(24) $$\begin{array}{c} \frac{L_{irc}}{L_{ir}^{t}}=\frac{V_{ivc}}{V_{iv}^{t}},\;i\in T,\;\;r\in R,\;\;v\in O_{r},\;\;c\in C. \end{array}$$

Constraint (24) is reformulated as an equation involving bilinear terms,

(25) $$\begin{array}{c} V_{ivc}\cdot L_{ir}^{t}=L_{irc}\cdot V_{iv}^{t},\;i\in T,\;\;r\in R,\;\;v\in O_{r},\;\;c\in C. \end{array}$$

Note that constraint (25) is correct even when operation *v* is not assigned to priority-slot *i*, as then

(26) $$\begin{array}{c} V_{iv}^{t}=V_{ivc}=0. \end{array}$$

#### 2.5.14. Resource Constraints

Resource constraints restrict the use of resources throughout the scheduling horizon.

#### 2.5.15. Tank Capacity Constraint

The level of materials in the tank *r* must remain between minimum and maximum capacity limits  $\underline{L_{r}^{t}}$  and  $\overset{-}{L_{r}^{t}}$, respectively. Let *L*_0*r*_^*t*^ be the initial total level and let *L*_0*rc*_ be the initial level of crude *c* in the tank  *r*. As simultaneous charging and discharging of tanks is forbidden, the following constraints are sufficient:

(27) Lrt_≤Lirt≤Lrt−, i∈T,  r∈RS∪RC,0≤Lirc≤Lrt−, i∈T,  r∈RS∪RC,  c∈C,Lrt_≤L0rt+∑i∈T ∑v∈IrVivt−∑i∈T ∑v∈OrVivt≤Lrt−,r∈RS∪RC,0≤L0rc+∑i∈T ∑v∈IrVivc−∑i∈T ∑v∈OrVivc≤Lrt−,r∈RS∪RC,  c∈C.

#### 2.5.16. Demand Constraint

Demand constraints define lower and upper limits, $\underline{D_{r}}$  and $\overset{-}{D_{r}}$, on total volume of products transferred out of each charging tank *r* during the scheduling horizon,

(28) $$\begin{array}{c} \underline{D_{r}}\leq \sum_{i\in T\;}\sum_{v\in O_{r}}V_{iv}^{t}\leq \overset{-}{D_{r}},\;r\in R_{C}. \end{array}$$

## 3. Finite State Theory

This section presents in a somewhat informal way those basic notions and definitions from formal language and finite state theories, which are relevant for the sections to follow. Related definitions are taken from literature [20, 21]. Readers, who are unfamiliar with formal language theory, are advised to consult the sources whenever necessary.

### 3.1. Finite State Automata

A DFA is a 5-tuple (*Q*, Σ, *δ*, *i*, *F*), where *Q* is a set of states, Σ is an alphabet, *i* is the initial state, *F*⊆*Q* is a set of final states, and *δ* is a transition function mapping *Q* × Σ to *Q*. That is, for each state *u* and symbol *a*, there is at most one state that can be reached from *u* by “following” *a* (Figure 2).

### 3.2. Finite State Transducers

A finite state transducer (FST) is a 6-tuple (Σ_1_, Σ_2_, *Q*, *δ*, *i*, *F*), where *Q*, *i*, and *F* are the same as for DFA, Σ_1_ is input alphabet, Σ_2_ is output alphabet, and *δ* is a function mapping *Q* × (Σ_1_ ∪ {*ε*})×(Σ_2_ ∪ {*ε*}) to a subset of the power set of *Q* (Figure 3). Intuitively, an FST is much like an NFA except that transitions are made on strings instead of symbols and, in addition, they have outputs.

### 3.3. Finite State Calculus

As argued in Karttunen [22–25], many of the rules used can be analyzed as special cases of regular expressions. They extend the basic regular expression with new operators. These extensions make the finite state automation and finite state transducer become more suitable for particular applications. The system described below was implemented using FSA Utilities [26], a package for implementing and manipulating finite state automata, which provides possibilities for defining new regular expression operators. The part of FSAs built in regular expression syntax relevant to this paper is listed in Table 4.

One particular useful extension of the basic syntax of regular expressions is the replace-operator. Karttunen [22–25] argues that many phonological and morphological rules can be interpreted as rules which replace a certain portion of the input string. Although several implementations of the replace-operator are proposed, the most relevant case for our purposes is the so-called “leftmost longest-match” replacement. In case of overlapping rule targets in the input, this operator will replace the leftmost target, and in cases where a rule target contains a prefix which is also a potential target, the longer sequence will be replaced. Gerdemann and van Noord [27] implement leftmost longest-match replacement in FSA as the operator:

(29) $$\begin{array}{c} \text{replace}\;\;\left(\text{Target},\text{LeftContext},\text{RightContext}\right), \end{array}$$

where Target is a transducer defining the actual replacement and LeftContext and RightContext are regular expressions defining the left and right context of the rule, respectively. The segmentation task discussed in the mutation procedure makes crucial use of longest-match replacement.

## 4. The Hybrid Algorithm

From the point view of optimization efficiency and robustness, a novel two-level optimization framework based on finite state method and GA is proposed for the MINLP model in this section.

### 4.1. Two-Level Optimization Structure

As the foundation of the framework, a two-level optimization structure is introduced. Once all binary variables are fixed the original problem becomes a relatively simpler model with only continuous variable. Following this deal, we rewrite (5) as follows:

(30) $$\begin{array}{c} \max\left(J\left(\xi ,z\right)\right)⟺\underset{z}{\max}\left[\underset{\xi }{\max}J\left(\xi ,\overset{-}{z}\right)\right], \end{array}$$

where *ξ* and *z* represent continuous and binary variables, respectively. Equation (30) shows when *z* is fixed as $\overset{-}{z}$, the submodel $J(\xi ,\overset{-}{z})$ can be solved optimally by continuous-optimization solvers in the inner level; then we update $\overset{-}{z}$ towards the best binary solution z* in the outer level.

We used an example in Figure 4 to show how binary solution can be mapped to a scheduling sequence. The schedule *S* = [7683513762] where 7 stands for the specific operation 7 to assign to position 1 corresponding to the binary decisions  *Z*_17_ = 1.

### 4.2. Initial Population

Based on the sequencing rules [18] and the extension to the regular expression calculus [22–25], a DFA model which builds legal schedules complying with sequencing rules and operation condition is constructed. The whole set of possible schedules is too huge to be processed at once. The DFA model of the schedule constitutes a reasonable framework, capturing all possible schedules and removing many redundant sequences of operations. Initial values of decision variables must satisfy the equality constraints and operation condition and therefore represent a feasible operating point.

Here, we still use the instance with 8 operations from Mouret et al. [18] to describe an efficient sequencing rule by using a regular expression. A feasible sequence *v*_1_ ⋯ *v*_*i*_ ⋯ *v*_*n*_ can be described by the following:

(31) $$\begin{array}{c} \text{sequence}=\left(\varepsilon +L_{a}\right){\left(L_{b}\cdot L_{a}\right)}^{*}\left(\varepsilon +L_{b}\right),\\ L_{a}=7\left(\varepsilon +4\right)\left(\varepsilon +6\right)\left(\varepsilon +1+14\right)\left(\varepsilon +2+26\right),\\ L_{b}=8\left(\varepsilon +3\right)\left(\varepsilon +5\right)\left(\varepsilon +1+13\right)\left(\varepsilon +2+25\right). \end{array}$$

However, this automation suffers from a serious problem of overgeneration. For example, the short length of the sequence may lead to infeasibility, while the long length of the sequence may result in an unsolvable model. It is an interesting challenge for finite state syntactic description to specify a sublanguage that contains all and only the sequences of valid length.

Our solution is to construct a suitable constraint for the sequences of valid length. The constraint expressions denote a language that admits sequences of valid length but excludes all others. We obtain the desired effect by intersecting the constraint language with the original language of sequence expressions. The intersection of the two languages contains all and only the valid dates:

(32) $$\begin{array}{c} \text{ValidSequence}=\text{Sequence}\cap \text{ValidLength.} \end{array}$$

The ValidLength constraint is a language that includes all sequences of length *n*:

(33) $$\begin{array}{c} \text{ValidLength}={\left(1+2+3+4+5+6+7+8\right)}^{n}. \end{array}$$

We have now completed the task of describing the language of valid sequences from the set of possible sequence expressions. It is also possible to create an automation on the basis of the regular expression and ValidSequence and then generate all possible sequences *v*_1_ ⋯ *v*_*i*_ ⋯ *v*_*n*_ accepted by the automaton. The processes are implemented using FSA Utilities [26] that is a package for implementing and manipulating DFA and finite state transducer. In order to generate all possible sequences. When all possible sequences *v*_1_ ⋯ *v*_*i*_ ⋯ *v*_*n*_ accepted by the automaton are generated, and the population of the according possible binary decisions is generated. In the initial population stage of GA, the population size is the number of individuals. When the number of individuals is given, a population of candidate solutions is generated by randomly selecting from the population of the all possible binary decisions.

### 4.3. Rule-Based Mutation Approach

In the mutation stage, we use a finite state transducer for this rule-based mutation process. The rule-based mutation strategy must obey the sequencing rule and the nonoverlapping constraint such that all involved solutions in GA are feasible.

The proposed mutation approach is a two-step procedure.

**Step 1**

Segmentation of the input sequence into a set of subsequences (i.e., the subsequence which belongs to the regular language L7 or L8).

**Step 2**

Mutation of the subsequences into others.

Formally, the rule-based mutation procedure is implemented as the composition of three transducers (see Algorithm 1).

An example of mutation including the intermediate steps is given for the sequence “7681325712” as shown in Figure 5.

#### 4.3.1. Segmentation Transducer

Segmentation transducer splits an input sequence into subsequences. The goal of segmentation is to provide a convenient representation level for the next mutation step.

Segmentation is defined as shown in Algorithm 2.

The macro “SSequence” defines the set of subsequences. The subsequences which belong to the regular language L7 and L8 are displayed in Tables 1 and 2. Segmentation attaches the marker “–” to each subsequence. The Targets are identified using leftmost longest-match, and thus at each point in the input, only the longest valid segment is marked.

#### 4.3.2. The Mutation Rules

In the GA process, the mutation rules are made by carefully considering nonoverlapping constraint between operations. A concrete instance for partially illustrating the mutation rules is given in Algorithm 3. Note that the final element of the left-context must be a marker and the target itself ends in “–.” This ensures that mutation rules cannot apply to the same subsequence.

## 5. Experimental Study

**Figure 1:**
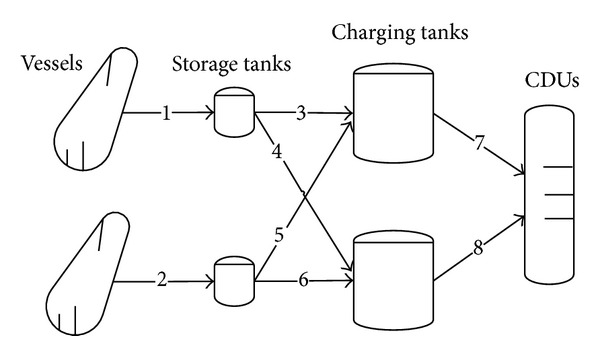
Crude oil operations system for the problem.

**Figure 2:**
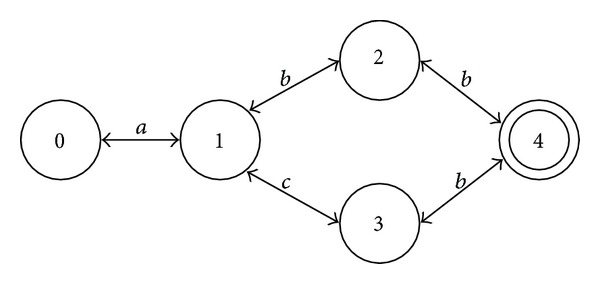
A deterministic finite state automaton (DFA).

**Figure 3:**
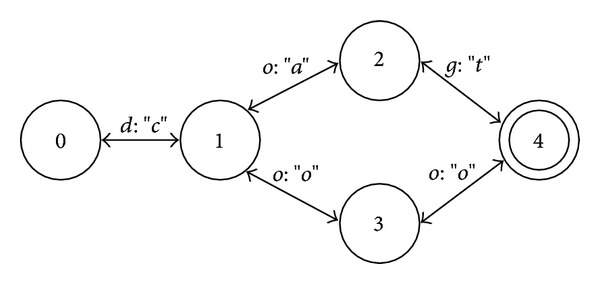
Finite state transducer encoding the relation {(dog, cat), (dog, cow)}.

**Figure 4:**
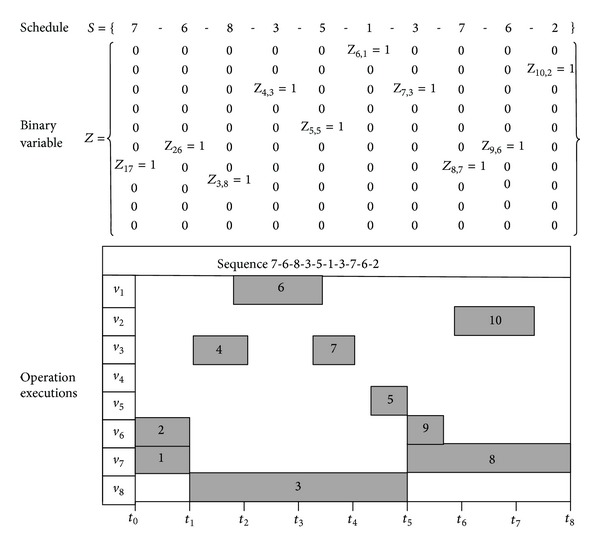
An example to indicate the relationship between binary variable and schedule.

**Figure 5:**
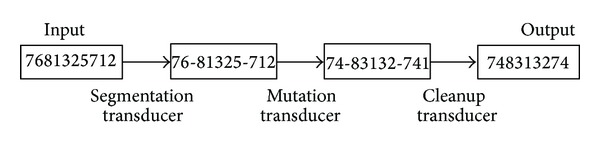
An example of mutation.

In this section, the same problem from the literature [18] is used for computational experiments. The proposed methodology is compared with existing promising algorithms, mixed-coding GA [15, 28].  Figure 1 depicts the refinery configuration for problem. The data involved in the problem are given in Table 3. The performance comparison with different computing times, such as 350 s, 500 s,…, 2400 s, is conducted. The objective value is used to statistically analyze the optimization results.

**Figure 6:**
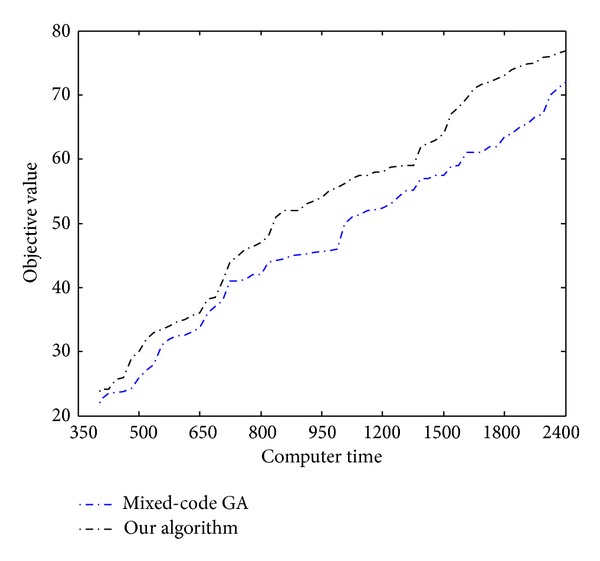
Average objective values of two methodologies.

The performance comparison between the two methodologies used is illustrated in Figure 6, which shows that the hybrid optimization algorithm which combined the finite state method and GA will statistically outperform the mixed-coding counterpart. The genetic algorithm which combined the finite state method and GA finds feasible solutions very fast and is able to find better solutions in reasonable time.

**Figure 7:**
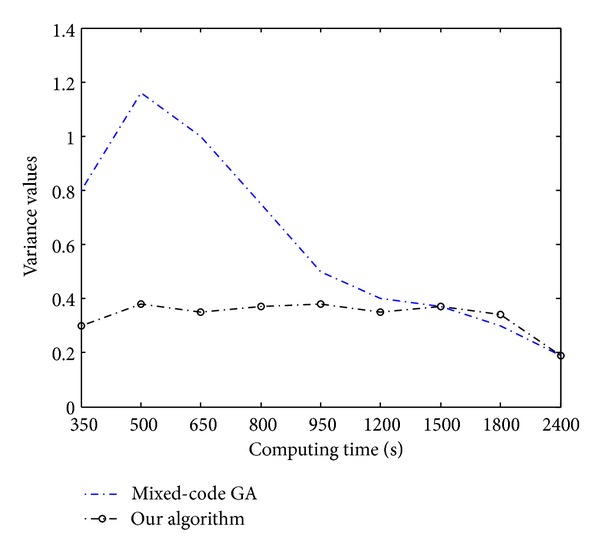
Variance values of two methodologies.

In Figure 7, we compare the objective variance of each iteration in the two evolution processes of these two kinds of methodology. By tracking the evolution process, we find that the mixed-coding GA is easy to stick in a local minimal sequence solution. This situation only can be improved through increasing the mutation scaling factor. However, this may result in a hard convergence, unless sufficient iterations are implemented. As for the hybrid optimization algorithm, the optimization processes of binary variable and continuous variable are separated. The performance of the whole methodology mainly depends on the FSM which captures most promising schedules and removes many redundant sequences of operations, so that the user can use a small population size of corresponding discrete variables to obtain suboptimal solutions. From Figure 7, we see that the proposed method has converged at 350 iterations as opposed to 2400 iterations for the mixed-coding GA.

The success of the proposed algorithm lies in a comprehensive analysis of the region of the search space and its capacity to focus the search on the regions with the partial solution. One of the good merits of the hybrid algorithm is that each solution involved in the GA algorithm is guaranteed to be feasible by using the mutation rules generated by DFM method while in existing GA algorithms the procedure to generate feasible solution under complex process constraints is very time costive. The deterministic finite automata (DFA) can easily represent this kind of structure. Furthermore, the complex process constraints can be very difficult to express with mixed integer programming. Consequently, it is unfeasible to solve the industrial problem by using MIP solver.

## 6. Conclusion

In this paper, a novel hybrid optimization algorithm which combined the finite state method and GA is proposed. The proposed algorithm constitutes a reasonable framework, capturing both the operating condition and sequencing rule of the schedule. The solution captures all possible schedules and removes many redundant sequences of operations. The algorithm is equivalent to introducing new structure information into the optimization process, which will help reduce the risk of trapping in a local minimal sequence solution. The hybrid optimization algorithm is an effective and robust tool to solve the crude oil scheduling problem in terms of efficiency and reliability. Algorithms only with the two properties are suitable for solving practical engineering application.

## Figures and Tables

**Algorithm 1:**
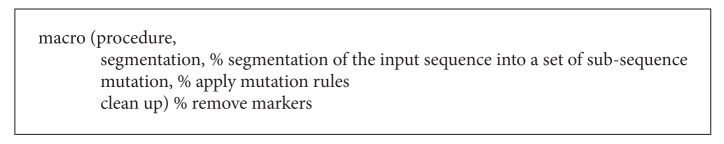


**Algorithm 2:**
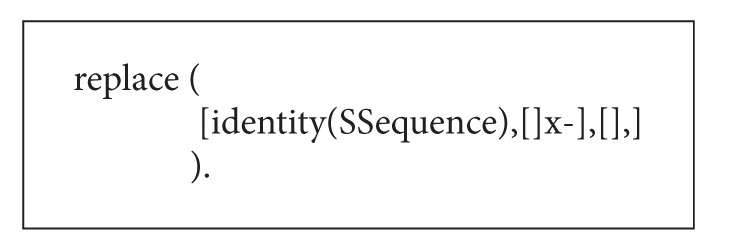


**Algorithm 3:**
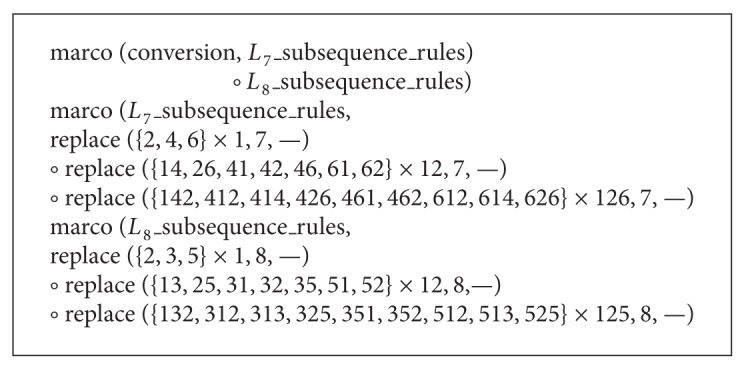
An example to demonstrate the mutation rules.

**Table 1:** Subsequence belonging to L_7_.

Length	Sequences belonging to *L*_7_									
1	7									
2	71	72	74	76						
3	712	714	726	741	742	746	761	762		
4	7126	7142	7412	7414	7426	7461	7462	7612	7614	7626
5	71426	74126	74142	74612	74614	74626	76126	76142		
6	741426	746126	746142	761426						
7	7461426									

**Table 2:** Subsequence belonging to L_8_.

Length	Sequences belonging to *L*_8_									
1	8									
2	81	82	83	85						
3	812	813	825	831	832	835	851	852		
4	8125	8132	8312	8313	8325	8351	8352	8512	8513	8525
5	81325	83125	83132	83512	83513	83525	85132	85125		
6	831325	835125	835132	851325						
7	8351325									

**Table 3:** Problem data.

Scheduling horizon			8 days		
Vessels	Arrival time		Composition	Amount of crude	

Vessel 1	0		100% A	1000	
Vessel 2	4		100% B	1000	

Storage tanks	Capacity		Initial composition	Initial amount	

Tank 1	[0,1000]		100% A	250	
Tank 2	[0,1000]		100% B	750	

Charging tanks	Capacity		Initial composition	Initial amount	

Tank 1 (mix X)	[0,1000]		100% C	500	
Tank 1 (mix X)	[0,1000]		100% D	500	

Crudes	1	Gross margin	Crude mixtures	Property1	Demand

Crude A	0.01	9	Crude mix X	[0.015,0.025]	[1000,1000]
Crude B	0.06	4	Crude mix Y	[0.045,0.055]	[1000,1000]
Crude C	0.02	8	Unloading flow rate	[0,500]	
Crude D	0.05	5	transfer flow rate	[0,500]	

**Table 4:** A fragment of FSA regular expression syntax and *U* transducers, and *R* can be either.

[]:	The empty string
[*R*_1_,…, *R*_*n*_]:	Concatenation
{*R*_1_,…, *R*_*n*_}	Disjunction
*R* ^Λ^:	Optionality
Identity (*A*):	Identity: the transducer which maps each element in *A* onto itself
*T*∘*U*:	Composition of the transducers *T* and *U*
macro (Term, *R*):	Use term as an abbreviation for *R*
